# Design optimization platform for assistive wearable devices applied to a knee damper exoskeleton

**DOI:** 10.1017/wtc.2025.10016

**Published:** 2025-07-10

**Authors:** Asghar Mahmoudi, Stephan Rinderknecht, Andre Seyfarth, Maziar A. Sharbafi

**Affiliations:** 1 Institute for Mechatronic Systems, Faculty of Mechanical Engineering, Technical University of Darmstadt, Darmstadt, Germany; 2 Lauflabor Locomotion Lab, Institute of Sports Science, Technical University of Darmstadt, Darmstadt, Germany

**Keywords:** optimisation, exoskeletons, design, biomechanics, human motor control

## Abstract

Designing optimal assistive wearable devices is a complex task, often addressed using human-in-the-loop optimization and biomechanical modeling approaches. However, as the number of design parameters increases, the growing complexity and dimensionality of the design space make identifying optimal solutions more challenging. Predictive simulation, which models movement without relying on experimental data, provides a powerful tool for anticipating the effects of assistive devices on the human body and guiding the design process. This study aims to introduce a design optimization platform that leverages predictive simulation of movement to identify the optimal parameters for assistive wearable devices. The proposed approach is specifically capable of dealing with the challenges posed by high-dimensional design spaces. The proposed framework employs a two-layered optimization approach, with the inner loop solving the predictive simulation of movement and the outer loop identifying the optimal design parameters of the device. It is utilized for designing a knee exoskeleton with a damper to assist level-ground and downhill gait, achieving a significant reduction in normalized knee load peak value by 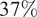 for level-ground and by 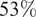 for downhill walking, along with a decrease in the cost of transport. The results indicate that the optimal device applies damping torques to the knee joint during the Stance phase of both movement scenarios, with different optimal damping coefficients. The optimization framework also demonstrates its capability to reliably and efficiently identify the optimal solution. It offers valuable insight for the initial design of assistive wearable devices and supports designers in efficiently determining the optimal parameter set.

## 1. Introduction

Optimizing the design of assistive wearable devices is crucial for enhancing their usability, effectiveness, and adoption (Shore et al., 2018). The optimal design of these devices’ mechanical and control parameters can be achieved by developing prototypes and testing them through systematic experiments involving human participants (Zhang et al., 2017). This Human-in-the-Loop Optimization (HILO) process, however, is inherently time-consuming and physically demanding for participants, often making it tiresome and costly. Furthermore, the results are typically limited to specific gait conditions, making generalization to diverse real-world scenarios challenging. Adjusting design parameters during experiments also requires emulators, which, despite their flexibility, are expensive and fail to fully replicate the final product (Slade et al., 2024). To address these limitations, biomechanical models are used as a viable alternative (Firouzi et al., 2025; Mahdian et al., 2023).

On the one hand, the inverse dynamics method is a widely used approach in biomechanical modeling for designing wearable assistive devices (Zhou et al., 2017). In this approach, the experimentally measured movement parameters taken from motion capture systems and Ground Reaction Forces (GRF) are used as the inputs to the model to determine the joint angles, muscle forces, and joint torques (Firouzi et al., 2021; Naseri et al., 2022). For instance, Marconi et al. employed inverse dynamics to evaluate the effects of weight distribution of powered ankle-foot orthoses on muscle activation and forces, assuming user kinematics remain unchanged by the device (Marconi et al., 2023). However, this assumption is a major limitation of the inverse dynamics method.

On the other hand, forward dynamics approaches do not require experimental measurements and rely solely on the neural controllers as inputs to the neuromuscular model to perform predictive simulation of movement (De Groote and Falisse, 2021; Febrer-Nafría et al., 2023). This method has been employed to optimize exoskeleton designs. For example, Bianco et al. demonstrated that multi-joint torque assistance yields 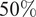 greater metabolic savings than single-joint devices (Bianco et al., 2022). Ratnakumar et al. used OpenSim Moco software to identify hip joint assistance as the most effective single-joint assistance case (Dembia et al., 2020; Ratnakumar et al., 2022), while Jin et al. proposed a framework based on Covariance Matrix Adaptation Evolutionary Strategy (CMA-ES) for predictive simulation of reflex-based models with exoskeleton interaction (Jin et al., 2024). SCONE software (Geijtenbeek, 2019), also using the CMA-ES method, has been utilized for diverse applications, including optimal torque assistance for hip exoskeletons (Ratnakumar and Zhou, 2021), investigating ankle-foot orthosis stiffness for patients with muscle weakness (Waterval et al., 2023), and analyzing actuation mechanisms for balance recovery (Jabeen et al., 2023). Additionally, predictive simulation has been applied to design assistance modes for sloped walking (Li et al., 2020). While forward dynamics simulations offer a powerful tool for exploring the design space of assistive devices without the need for extensive experimental data, it is important to acknowledge the potential for discrepancies between simulated results and real-world performance. As highlighted by Franks et al. (2020), the translation from simulated assistance strategies to actual device performance can present significant challenges. They illustrated that the expected metabolic savings for the optimal device controller settings showed a similar trend between the experiment and simulation results, but the metabolic cost reductions were greatly overestimated in simulations. However, recent study by Jin et al. (2024) has demonstrated promising agreement between forward dynamics simulations and experimental data in the context of exoskeleton design, suggesting increased reliability of these methods. Similarly, Drewing et al. (2024) have shown improvement in some muscle activation predictions in comparison between AI-optimized control parameters using forward simulation and an empirical controller in follow-up experiments of assisted walking with an exosuit.

In the mentioned studies, the predictive simulation problem is solved for a predefined set of design parameters, and the best solution is selected from the tested cases. However, as the dimensionality of the design space increases, the number of candidate solutions grow exponentially, significantly increasing computational cost. Advanced optimization methods can mitigate this issue by efficiently navigating high-dimensional design spaces within a defined range of parameters to identify optimal solutions. Therefore, we propose an optimization framework that leverages predictive simulation and advanced optimization techniques to efficiently explore high-dimensional design spaces and identify optimal assistive device configurations. In this article, the design of a passive knee exoskeleton with a damping characteristic and a specific activation timing is optimized.

Optimizing the design of assistive wearable devices, such as knee exoskeletons, is essential to enhance their functionality and adaptability. The knee joint plays a crucial role in maintaining stability, absorbing shocks, supporting body weight, and assisting in swing motion (Amer et al., 2020; Zhang et al., 2020). It is also quite a vulnerable joint, susceptible to injuries, impairments, and conditions like osteoarthritis, spinal cord injury, and stroke (Flandry and Hommel, 2011). Knee exoskeletons aim to support individuals with these impairments (Wu et al., 2023) or augment healthy users’ locomotion by reducing metabolic cost, muscle fatigue, and improving load-carrying capacity (Shamaei et al., 2014; Tucker et al., 2017). Passive and semi-active exoskeletons often incorporate dampers at the knee joint to provide controllable resistance, enhancing energy efficiency during braking and enabling regenerative braking (Ma et al., 2017; Auberger et al., 2020). These dampers help mitigate excessive vibrations and shocks, and reduce the forces transmitted to the joint (Alvarado-Rivera et al., 2022; Andoh and Huang, 2023). Notably, the application of damping forces during the Stance phase, when the knee exhibits negative joint power, effectively dissipates energy and reduces the biological joint’s negative power (Xie et al., 2019; Zhang et al., 2020). This functionality is particularly advantageous during downhill walking, which increases negative power, negative work, and joint forces compared to level-ground gait (Kuster et al., 1995; Alexander and Schwameder, 2016; Masayuki et al., 2018; Montgomery and Grabowski, 2018; Nuckols et al., 2020). Additionally, varying damping characteristics based on the user’s physical attributes and specific movement activities, such as stair ascent or descent and sit-to-stand transitions, is critical for optimizing the exoskeleton’s performance across diverse conditions (Amer et al., 2020).

The primary objective of this study is to present a novel two-level optimization framework for designing exoskeletons based on predictive simulation of human movement. It addresses the key limitations of existing methods, particularly their difficulty in navigating high-dimensional design spaces by using an advanced optimization method. The framework identifies the optimal physical and control design parameters in the outer loop and solves the predictive simulation of movement in the inner loop. To illustrate its efficacy, we applied the framework to the design of a knee exoskeleton with a damper, aimed at assisting level-ground and downhill gait in able-bodied users. Specifically, the optimization determines the optimal damping coefficient and the phases of the gait cycle in which the damper should be engaged. We hypothesize that applying a damping torque to the knee joint will reduce both knee load and the negative knee power peak, consistent with established knee biomechanics during gait and previous findings on knee damper exoskeletons (Xie et al., 2019; Zhang et al., 2020). Furthermore, we expect the optimal damping coefficient for level-ground and sloped gait to differ, which highlights the importance of incorporating a variable damper in the device for different movement scenarios.

The remainder of this article is structured as follows: Section 2 details the methodology, beginning with an overview of the proposed optimization framework (Section 2.1), followed by the formulation of the passive knee exoskeleton design problem (Section 2.2), and concluding with the description of validation and performance evaluation methods (Section 2.3). Section 3 presents the results, starting with the baseline gait simulations (Section 3.1) and then discussing the optimization outcomes and their effects on biomechanics (Section 3.2). Section 4 provides a discussion of the methodology validation and the optimal exoskeleton design (Sections 4.1 and 4.2), along with the limitations of the study and potential future research directions (Section 4.3). Finally, Section 5 concludes the article by summarizing the key findings and contributions.

## 2. Methods

### 2.1. Overview of the optimization framework

**Figure 1.**
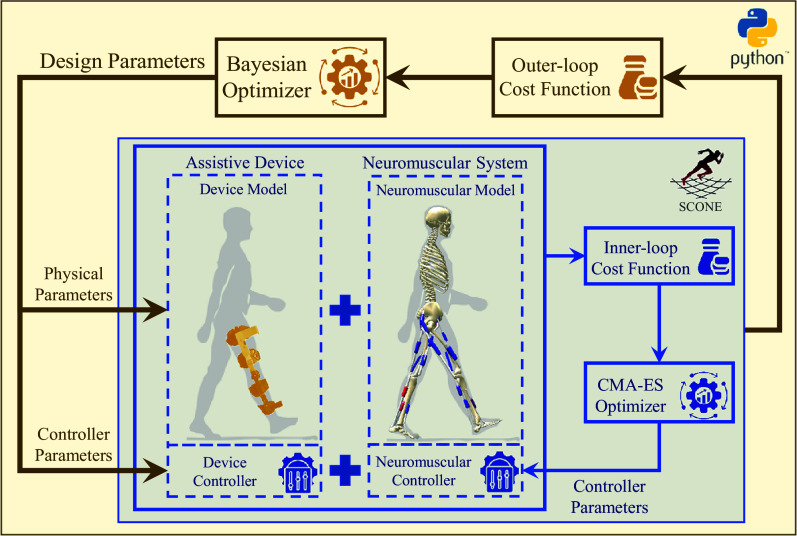
Overview of the optimization framework for designing assistive wearable devices. The inner loop, implemented in the SCONE software, solves the predictive simulation of movement and includes the device and neuromuscular models and controllers, a cost function, and the CMA-ES optimizer for optimizing the neuromuscular controller parameters. The outer loop, implemented in Python, employs a Bayesian optimizer to identify the optimal design parameters of the assistive device by minimizing a cost function derived from the inner loop’s simulation results.

The overview and important components of the proposed optimization framework are illustrated in Figure 1. It is a two-level modular optimization platform developed in Python. Its aim is to find the optimal design parameters of an assistive device by minimizing a given cost function. The platform consists of two nested optimization loops. The outer loop optimizes the design parameters of the assistive device, and the inner loop solves the predictive simulation of movement.

The inner optimization loop solves the predictive simulation of movement using the SCONE software, with the high fidelity dynamics (Hyfydy) simulation engine (Geijtenbeek, 2021) employed to accelerate forward dynamics computations. It comprises three key components: (1) a combined neuromuscular model of the human and a model of the assistive device, linked via interaction dynamics; (2) a neuromuscular feedback controller for the human model and a device controller; and (3) a cost function derived from solving the forward dynamics equations. The inner loop optimizes the neuromuscular controller parameters using the CMA-ES algorithm.

The outer loop optimizes the assistive device’s design parameters using Bayesian Optimization, implemented via the Scikit-Optimize library in Python Scikit-Optimize Contributors (2024). This method is well-suited for noisy, expensive to evaluate, and black-box cost functions (Garnett, 2023). A Gaussian process models the objective function with a Matern kernel, with hyperparameters (kernel length scales, covariance amplitude, and noise level) tuned during optimization. After initializing with a predefined number of random evaluations, the optimizer generates new parameter sets, continuing until either convergence or a maximum iteration limit is reached. Convergence is defined by minimal improvement over the best cost value in the last 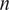 iterations. At each step, the acquisition function, chosen probabilistically from the lower confidence bound, expected improvement, or probability of improvement functions, guides the search Scikit-Optimize (2024). The outer loop thus minimizes the cost function derived from the inner loop’s predictive simulation results for both the assistive device’s physical and control parameters.

### 2.2. Design of a passive knee exoskeleton

To demonstrate the performance of the proposed design framework, a passive knee exoskeleton is simulated to assist able-bodied users during level-ground and downhill gait. Predictive simulations of both movement scenarios without the exoskeleton are conducted in SCONE and used as baseline conditions. The validity of the baseline simulations is qualitatively assessed by comparison with experimental data, which is presented in the next section.

A two-dimensional sagittal plane musculoskeletal model (*H0918v3.hfd*) is used for the predictive simulation of both baseline and assisted conditions. The model includes 9 degrees of freedom (pelvis tilt, pelvis translation in the x and y directions, right and left hip flexion, knee, and ankle angles) and 18 muscles (right and left hamstrings, biceps femoris short-head, gluteus maximus, iliopsoas, rectus femoris, vasti, gastrocnemius, soleus, and tibialis anterior). The muscles are Hill-type musculotendon units configured according to Delp et al. (1990) with the modified version of the muscle dynamics from Millard et al. (2013) to enhance performance in the Hyfydy solver. Two contact spheres per foot provide linear damped spring contact forces. For downhill gait, the ground is tilted by 5 degrees clockwise relative to the level-ground condition. A modified reflex-based controller from Geyer and Herr (2010) governs muscle activation, validated for predictive simulations of level-ground and inclined gait (Dorn et al., 2015; Li et al., 2020). The cost function used to optimize the parameters of the SCONE model consists of four terms:(1)

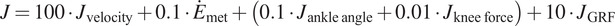



where (1) 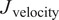 is a penalty term that forces the model to move with the predefined speed of 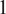 ( 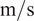 ), (2) 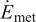 is a measure for the estimated metabolic cost based on Wang et al. (2010), (3) 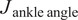 and 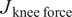 are the ankle joint position and the knee joint force penalties, and (4) 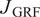 is a measure that penalizes the normalized GRFs above 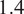 . A CMA-ES optimizer solves the predictive simulation over a 10-second duration. The optimization seeks the optimal values for 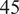 neuromuscular controller parameters, 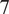 joint offset terms for degrees of freedom, and 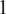 stance load threshold parameter used to identify the Stance phase.

**Figure 2.**
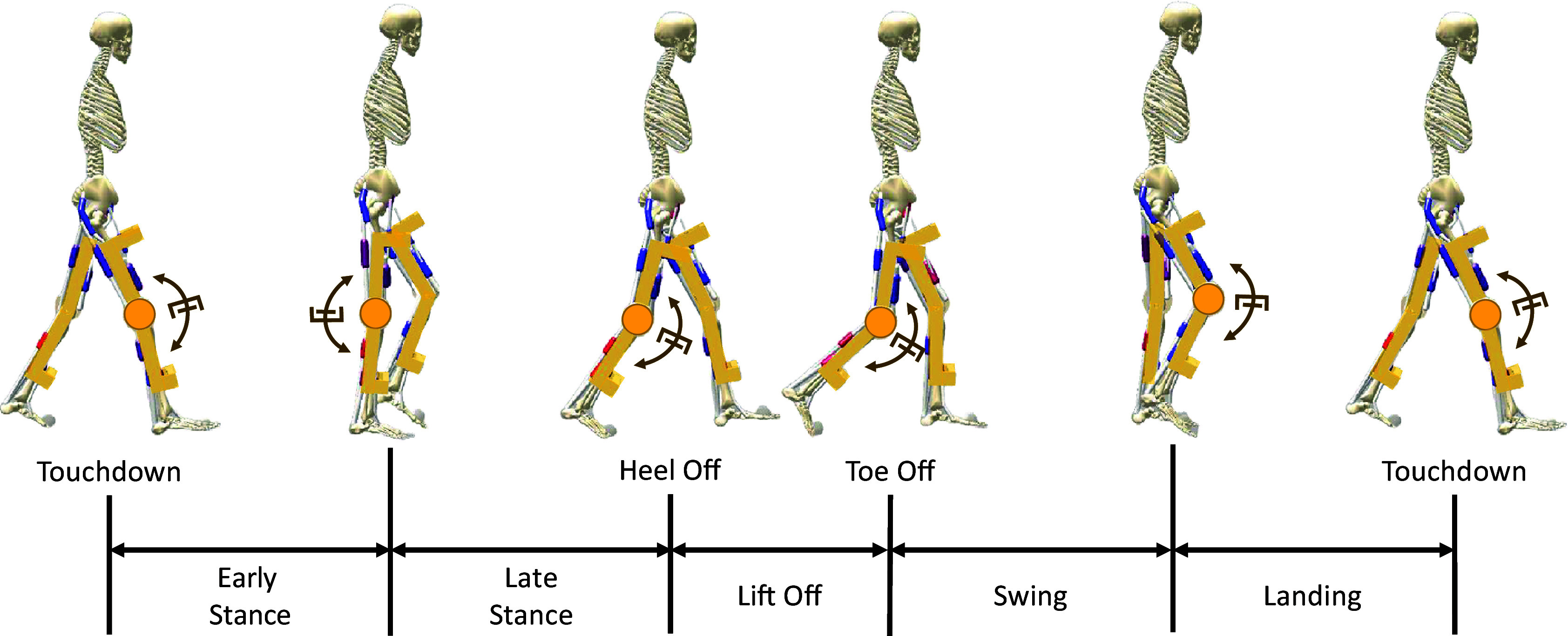
Illustration of the gait cycle divided into five phases based on SCONE definitions Scone Software (2024): Early Stance, Late Stance, Lift Off, Swing, and Landing. Each phase is depicted by a still photo of the neuromuscular model at the onset of the corresponding phase. The knee exoskeleton structure, adapted from a previously developed design (Mahmoudi et al., 2025), is integrated into the neuromuscular model. Unlike the original design, which featured a Pneumatic Artificial Muscle (PAM) as its actuator, this exoskeleton incorporates a damper and clutch mechanism at the knee joint. The damper, highlighted in the figure, applies resistive torques during specific gait phases.

Additionally, the exoskeleton’s model and controller are integrated into the neuromuscular model of both legs. The structural components of the exoskeleton, specifically the thigh ( 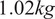 ) and shank ( 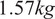 ) sections, are modeled based on the center of mass (COM), mass, and inertia derived from CAD files of a previously developed and tested knee exoskeleton (Mahmoudi et al., 2025) (as is seen in Figure 2). While this model replicates the physical structure, it does not include the damper and clutch mechanism of the device designed in this study. As such, potential effects of modifications to segment sizes and weights are not considered in the simulation. The optimization of the neuromuscular controller for assisted conditions begins with the mean and standard deviation (std) values obtained from the baseline simulation. Given that these std values are relatively small, this approach ensures minimal deviation from the optimal parameters of the baseline gait. Even slight variations in the device parameters can cause significant deviations in the optimal parameter solutions, as highlighted by Firouzi et al. (2024). This constraint helps avoid large deviations of the optimized parameters from the baseline values during assisted gait.

The designed knee exoskeleton is a passive device with a linear rotational damper, engaged during specific phases of the gait cycle via a clutch mechanism. The gait cycle is divided into five phases as defined in SCONE: Early Stance, Late Stance, Lift Off, Swing, and Landing (Figure 2). Transitions between phases are defined by the sagittal foot position and the normalized leg load. The Landing phase transitions to Early Stance when the leg load exceeds the stance load threshold. Early Stance progresses to Late Stance when the sagittal foot position falls below the Late Stance threshold. Late Stance transitions to Lift Off when the contra-lateral leg load surpasses the stance load threshold or when the sagittal position drops below the liftoff threshold. Lift Off transitions to Swing when the leg load falls below the Swing load threshold. Finally, Swing transitions to Landing when the sagittal foot position exceeds the landing threshold, completing the gait cycle Scone Software (2024). The damper is engaged only during selected phases, applying zero torques during the disengaged phases. The resisting torque applied to the neuromuscular model’s knee joint is:(2)

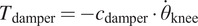



where 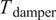 is the exoskeleton torque, 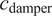 is the damping coefficient, and 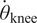 is the angular velocity of the knee joint. The damping coefficient 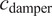 and the clutch activation pattern are the two design parameters. The damping coefficient is a continuous parameter optimized within the range of 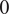 – 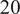 ( 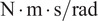 ), based on the maximum knee moment (approximately 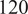 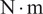 ) and maximum knee angular velocity (approximately 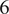 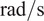 ) of the baseline simulation of a 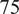 ( 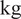 ) individual walking downhill at a 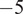 °slope. These upper ranges are consistent with experimental data (Kuster et al., 1995; Nuckols et al., 2020). The clutch activation pattern is a discrete parameter optimized across all 31 possible combinations of the five gait phases, determining when the damper resists the knee motion.

The outer loop cost function combines two terms: (1) Biological Knee Load, calculated as the mean of the top 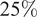 of knee load values, averaged across strides for both legs; and (2) Cost of Transport (COT), defined as the total metabolic cost (sum of estimated muscle metabolic costs) divided by the distance traveled. Both terms are normalized by division to their respective baseline values for level-ground and downhill gait. The final cost function is:(3)

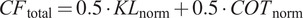



where 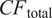 is the total cost function, 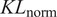 is the normalized knee load, and 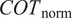 is the normalized cost of transport.

The Bayesian optimizer starts with 10 random evaluations and terminates if either the cost function improvement over the last 15 iterations is < 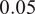 or 100 iterations are completed. A high cost value of 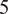 is assigned to distinguish unstable solutions. To reduce the risk of reaching a local minimum and improve the chance of global optimization, each problem is solved 5 times with different initial random seeds, and the best result is selected as the final design solution. All simulations are performed on a system with an Intel(R) Core(TM) i5-10400 CPU @ 2.90 GHz and 16 GB of RAM.

### 2.3. Validation methods

This section outlines the methods used to validate the predictive simulations and the performance of the optimization framework. A more detailed discussion and supporting evidence for the validation of the baseline simulations against experimental data are provided in Section 4.1. The predictive simulation results for level-ground and sloped gait in SCONE have been previously validated against experimental data (Li et al., 2020; Veerkamp et al., 2021). To further validate the accuracy of the baseline predictive simulations in this study, the kinematics and kinetics variables of the predicted movements are qualitatively matched with the changes observed in the experimental studies (Kuster et al., 1995; Alexander and Schwameder, 2016; Masayuki et al., 2018; Montgomery and Grabowski, 2018; Nuckols et al., 2020).

Moreover, the performance of the optimization framework is evaluated by comparing the results of the Bayesian optimizer with those obtained by exhaustively simulating the full design space at a fixed resolution for both gaits. More specifically, 41 damping values ( 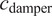 ) between 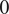 and 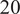 (step size: 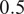 ) and all 31 possible clutch mechanism configurations are evaluated, resulting in 1271 unique parameter sets. Predictive simulations and cost functions for each set provide a detailed mapping of the design space, enabling an evaluation of the optimal solution. Additionally, to assess the framework’s robustness and its susceptibility to local minimum, the optimization is repeated with 20 additional different random seeds.

## 3. Results

This section presents the baseline simulation results for level-ground and downhill gait without the exoskeleton, followed by the optimization outcomes, including the performance evaluation of the optimization framework. Finally, we analyze the changes in kinematics, kinetics, metabolic cost, and muscle-tendon unit forces resulting from the addition of the exoskeleton.

### 3.1. Baseline simulations

**Figure 3.**
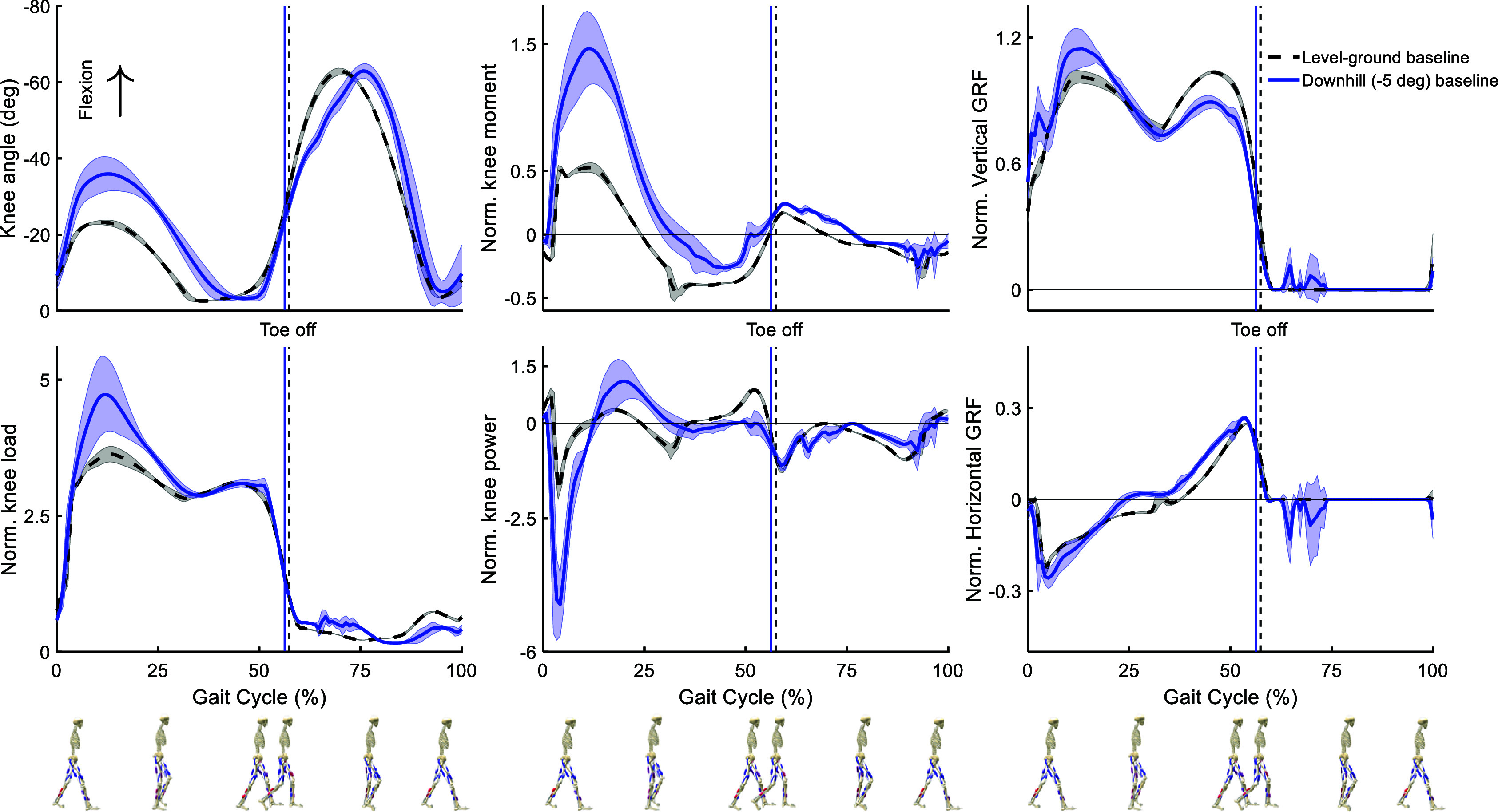
Kinematics and kinetics results of predictive simulation of baseline conditions (movement without exoskeleton) in level-ground (dashed black line) and downhill slope (solid blue line) gait. The results are normalized for each gait cycle (from touchdown of the right leg to the next touchdown of the same leg) and averaged over the gait cycles of one full movement for each condition. The shaded area around each curve indicates the standard deviation values. Stance and Swing phases are separated by the respective vertical lines, indicating the Toe Off events.

The quality of the forward simulation of baseline gait in the level-ground and downhill conditions can be evaluated in qualitative comparison to experimental findings (Kuster et al., 1995; Lay et al., 2006; Alexander and Schwameder, 2016; Dewolf et al., 2018; Masayuki et al., 2018), using the predicted kinematics and kinetics of movement. Figure 3 shows some of the most important differences observed between the two movement scenarios.

**Figure 4.**
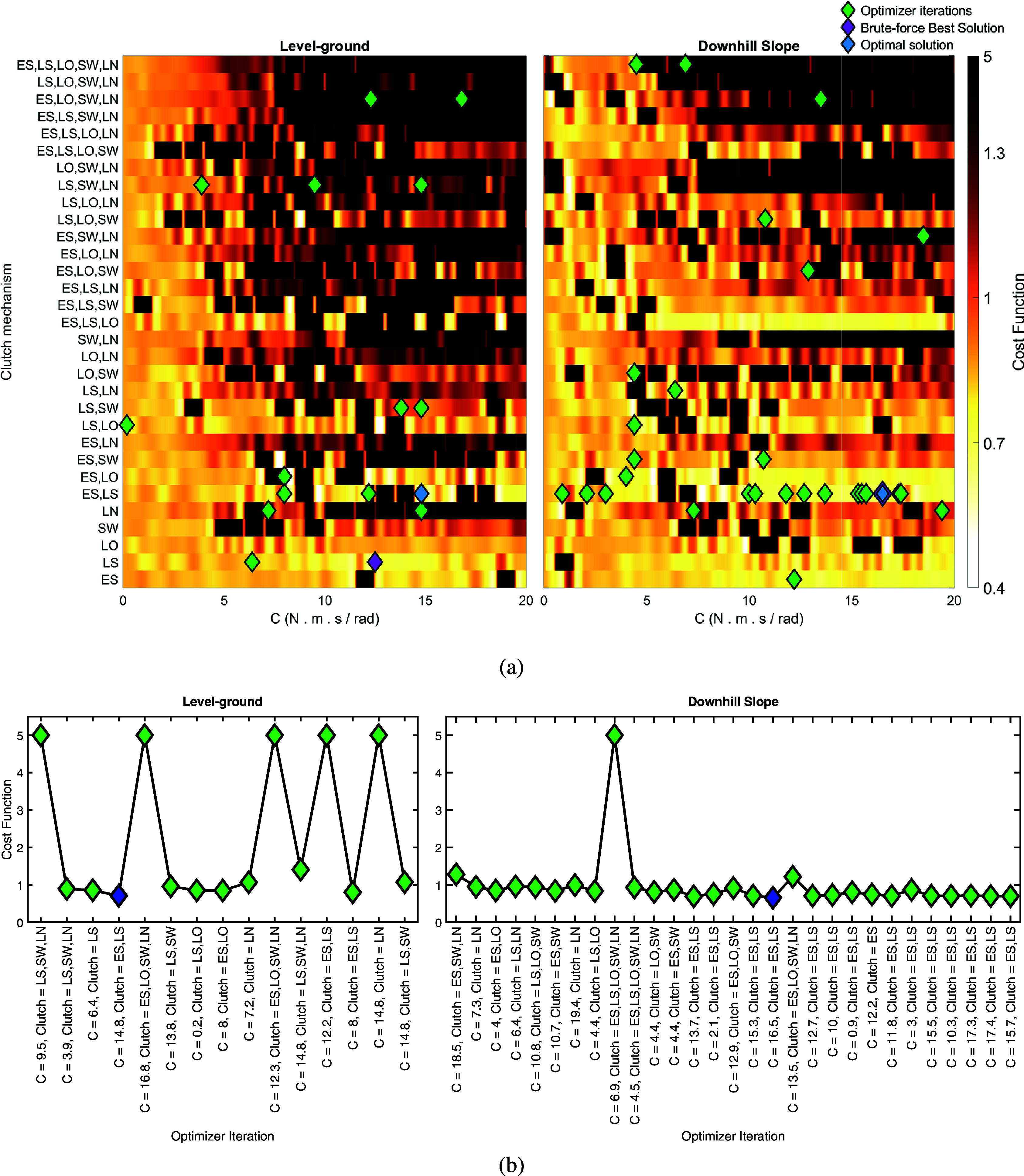
(a) Heat-map of the cost function values for the knee exoskeleton design in level-ground and downhill ( 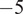 ) gait. The design space comprises the damping coefficient ( 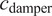 ) of the linear knee damper and the clutch mechanism that determines damper engagement across the five gait phases: Early Stance (ES), Late Stance (LS), Lift Off (LO), Swing (SW), and Landing (LN). The cost function, combining knee load and estimated cost of transport, is calculated for 1271 simulated design configurations, covering the entire design space. Green diamonds represent the iterations explored by the optimizer from the best of five optimization runs and blue diamonds indicate their optimal solutions. Purple diamonds mark the best solutions from the brute-force search of the entire design space. The best solution of the optimizer and the brute-force search in the downhill gait condition are the same. (b) The exploration order of the optimizer in each movement scenario. The abbreviations, shapes, and colors of indicated points are consistent with the heat-map.

The kinematics and kinetics of the knee joint show distinct trends during the downhill compared to the level-ground gait. It is observed that the knee flexion angle increases in downhill gait during the Stance phase, from approximately 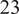 °to 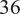 °, and remains relatively similar to the level-ground gait during the Swing phase. It can also be observed that the load on the knee joint increases during Early Stance in the downhill gait from the normalized peak value of 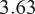 to 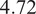 . The peaks of the normalized knee moment are fairly synced in the Early Stance phase while the downhill gait shows a higher positive peak ( 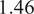 compared to 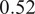 ) and a lower negative normalized peak value ( 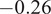 compared to 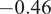 ). Similarly, the highest negative normalized knee power peak occurs in the Early Stance for both movements and shows a significant increase in the downhill walking ( 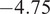 ) compared to the level-ground gait ( 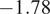 ). The positive power peak at the Early Stance shows an increase in the downhill gait (from 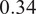 to 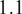 ), as well. The M-shaped vertical component of the GRF can be observed in both gait conditions. However, the downhill gait shows an increase in the first peak while the second peak decreases compared to the level-ground gait. Additionally, the GRF is non-zero during the early Swing phase in downhill gait, which indicates scuffing of the Swing leg in the simulated movement. The horizontal component of the GRF shows a similar trend for the two conditions.

### 3.2. Optimization results

In the best of five runs, the optimizer converges to the optimal solution in 15 iterations for level-ground gait and 28 iterations for downhill gait. Figure 4(a) shows heat-maps of the design space for the two exoskeleton parameters, generated from simulations across 1271 considered solutions. The order of exploration for the optimizer in the best run of the optimization platform is also presented in Figure 4(b). The optimal solution for both scenarios activates the clutch during Early and Late Stance phases, disengaging the damper during the other phases. The optimal damper coefficient 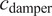 is 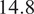 ( 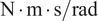 ) for the level-ground and 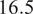 ( 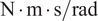 ) for the downhill gait, yielding optimized cost function values of 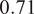 and 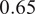 , respectively. The best condition in the brute-force investigation of all possible configurations resultes in the minimum cost function values of 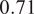 for the level-ground (with 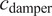 of 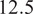 ( 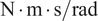 ) and active during the Late Stance phase) and 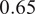 for the downhill gait (with 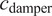 and clutch mechanism the same as the optimization result).

**Figure 5.**
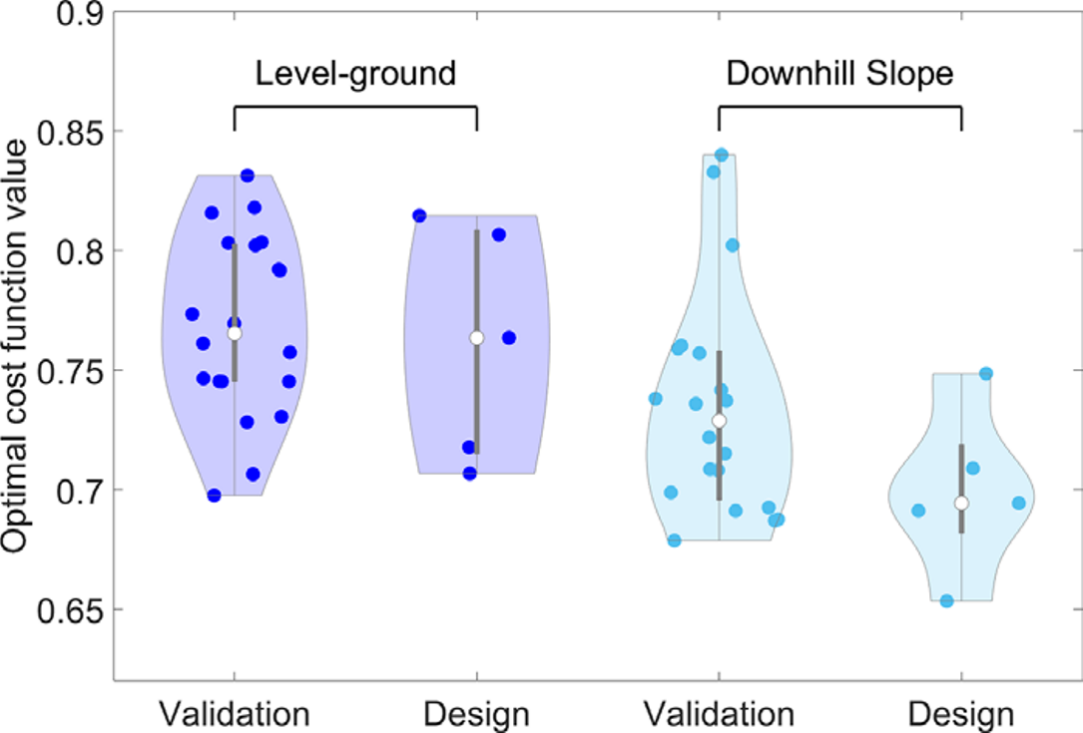
The median, 25th and 75th percentiles, maximum, minimum, and the kernel density estimate of the cost function values for the optimal results of multiple implementations of the optimization platform for the level-ground (first two graphs on the left side) and downhill (first two graphs on the right side) gait scenarios. The five runs of the optimizer used for the design of the device are also presented separately from the 20 runs used for investigation of the optimization platform’s performance.

Figure 5 presents the results of repeating the optimization process with different random initial seeds for the level-ground and downhill slope gait scenarios. The points in the Figure represent the cost function values of the optimal solutions in each implementation of the optimization process. The results of the five runs that are used for the design of the device are presented separately from the 20 runs that are used for verification of the optimizer performance. The results show that five runs of the optimization with different initial random seeds give a comparable optimal solution to the 20 runs of the optimizer. For the level-ground condition, the optimized cost function across 20 implementations has a median of 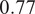 [ 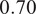 , 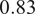 ] (median [min, max]), while the five implementations used for exoskeleton design show a median of 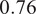 [ 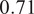 , 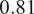 ]. In the downhill condition, the median values are 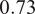 [ 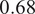 , 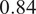 ] and 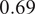 [ 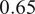 , 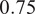 ] for the 20 and 5 implementations, respectively.

**Figure 6.**
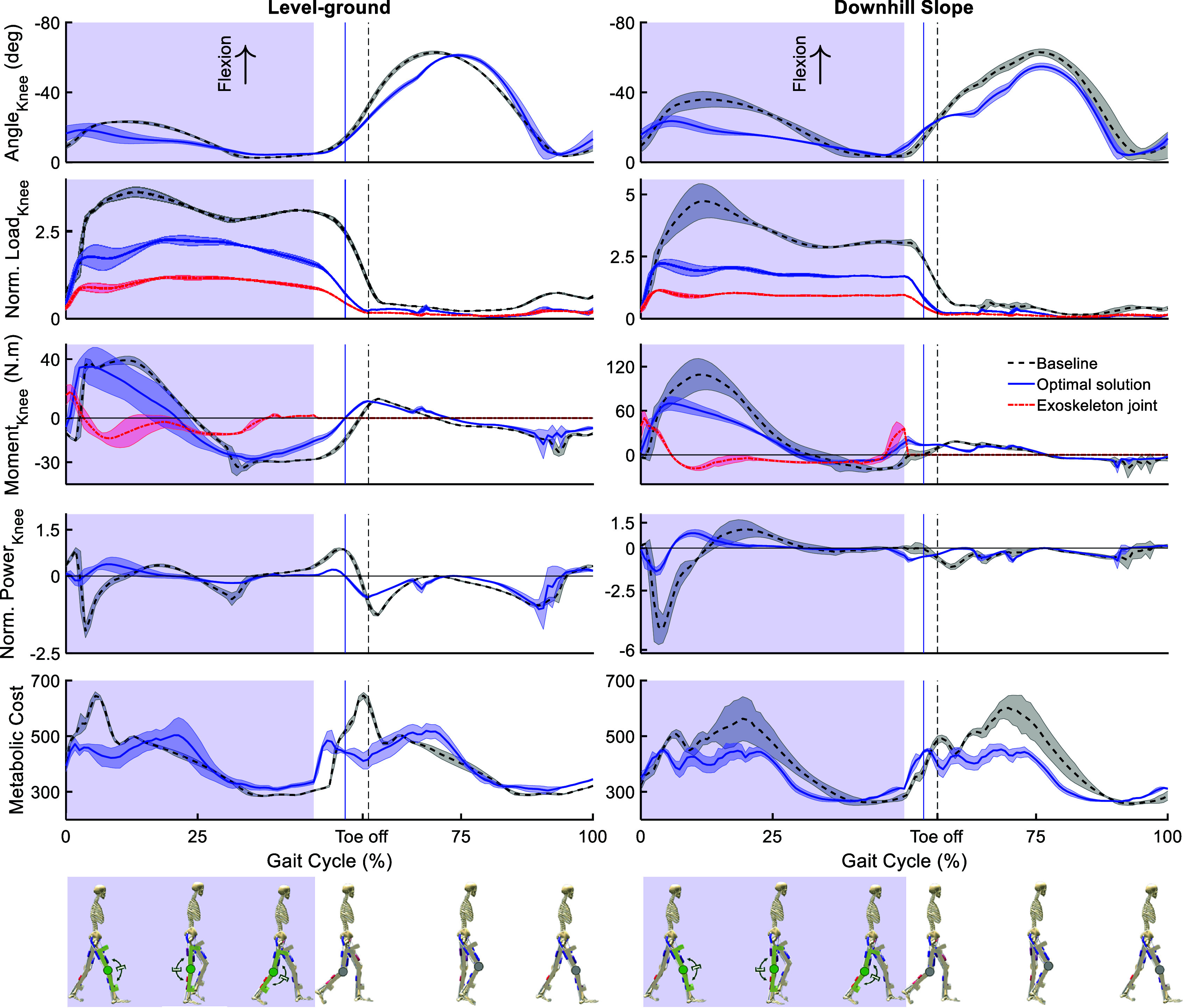
Knee kinematics, kinetics, and the metabolic cost of movement for the assisted scenario with the optimal knee exoskeleton design (solid blue lines) compared to the baseline condition without an exoskeleton (dashed black lines). The red dashed lines show the contribution of the exoskeleton joint. The results are normalized for each gait cycle (between two sequential touchdowns of the right leg) and averaged over all gait cycles of the entire movement for each condition. The shaded area around the lines indicates the standard deviation values. The Stance and Swing phases are separated by the respective vertical lines indicating the Toe Off event. The shaded blue area highlights the damper engagement phases.

Figure 6 illustrates the knee kinematics and kinetics, and the metabolic cost (an estimation of the metabolic cost of movement) for walking with the optimal exoskeleton design in the two movement scenarios compared to the baseline (no device) condition. The maximum flexion angle of the knee joint during the Stance phase decreases for both conditions, while the downhill gait scenario shows a more significant drop from 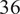 to 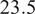 (around 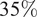 reduction). The results for both movement scenarios indicate a significant reduction of the knee load during the Stance phase. The normalized peak value drops by 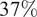 in the level-ground condition and by 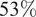 for the downhill slope condition. Additionally, the peak positive knee moment in the Early Stance phase has decreased for the downhill gait by around 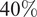 , whereas it stayed relatively similar for the level-ground gait. The maximum torque applied by the damper is 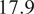 ( 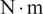 ) in the level-ground ( 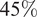 of the biological baseline value) and 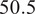 ( 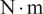 ) in the downhill gait ( 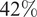 of the biological baseline value), both at the Early Stance phase directly following touchdown. Furthermore, the negative peak power of the knee joint during Early Stance has significantly reduced in both conditions from 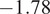 and 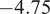 to 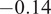 and 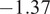 for the level-ground ( 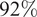 reduction) and downhill ( 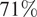 reduction) gaits, respectively. Moreover, the metabolic cost shows a decrease in the peak values when the device is added.

**Figure 7.**
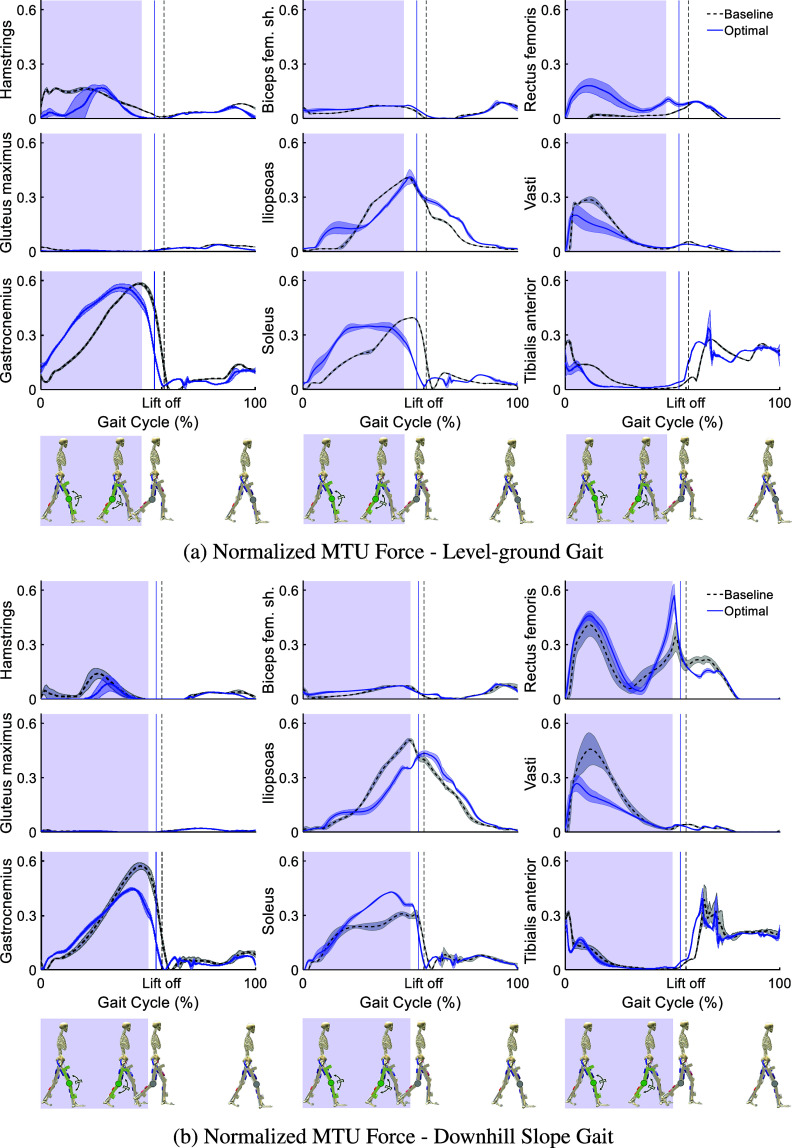
Normalized Muscle Tendon Unit (MTU) forces in the (a) level-ground, and (b) downhill slope gait, for the optimal knee exoskeleton design (solid blue lines) compared to the baseline condition, when the movement is simulated without an exoskeleton (dashed black lines). The results are normalized in each gait cycle (from touchdown of the right leg to the next touchdown of the same leg) and averaged over the gait cycles of one movement for each condition. The shaded area around the graphs illustrates the standard deviations. The Stance and Swing phases are separated by the respective vertical lines indicating the Toe-off events. The shaded area indicates the phases of the gait in which the damper is engaged.

Figure 7 compares normalized forces for the model’s nine muscles between baseline and optimal assisted conditions in level-ground and downhill walking, using a consistent y-axis scale for direct comparison. During level-ground walking, muscle force adaptation shows distinct patterns. Among knee extensors, the Vasti (monoarticular) significantly decreases, while the Rectus Femoris (biarticular) increases. For knee flexors, the Hamstrings (biarticular) reduces force, but the Gastrocnemius (biarticular) increases. The Biceps Femoris Shorthead (monoarticular) shows minimal force and change. Other muscles, including the Iliopsoas, Gluteus Maximus, and Tibialis Anterior, demonstrate reductions in peak forces during Stance, while the Soleus shows increased force. In downhill walking, the muscular response is generally characterized by force reductions or minimal changes for knee-crossing muscles. The Vasti, Hamstrings, and Gastrocnemius all exhibit decreased peak forces during Stance. Unlike level-ground, the Rectus Femoris force does not significantly increase, remaining similar to baseline. The Biceps Femoris Shorthead again displays minimal activity. These results indicate a complex, condition-dependent reorganization of muscle activity induced by the exoskeleton.

## 4. Discussion

### 4.1. Methodology validation

The kinematics and kinetics trends observed in the predictive simulation of baseline conditions align well with the results of experimental studies. The simulated results show a more bent knee angle in downhill walking compared to the flat terrain during the Stance phase (except the last 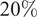 ), closely replicating the experimental results (Lay et al., 2006; Dewolf et al., 2018). Although, the increased knee flexion angle in the experimental studies persists until the end of the Stance phase. This agreement extends to the knee joint forces during sloped and level-ground walking, such as the rise in the first peak force during Early Stance (Alexander and Schwameder, 2016; Masayuki et al., 2018). An increase in the second peak of the knee load found in experimental studies is not predicted in our simulations, potentially due to the lower ground inclination and walking speed in our modeling. Correspondingly, the predicted increase in knee moment during the Stance phase, particularly in the first positive peak for downhill walking, agrees reasonably well with experimental data (Kuster et al., 1995; Lay et al., 2006; Masayuki et al., 2018). Additionally, the significant increase in the knee joint’s negative peak power during Early Stance for downhill walking, reported in the experimental studies (Kuster et al., 1995), is consistent with our simulated results. Finally, trends of GRF peak changes on sloped terrain versus flat terrain that match the experimental studies (Kuster et al., 1995; Lay et al., 2006) further validate the accuracy of our simulations in capturing the kinetic effects of environmental changes on locomotion. Overall, it can be concluded that the predictive simulations of baseline conditions presented in this study successfully capture the key kinematic and kinetic trends in level-ground and downhill gait with acceptable accuracy.

Repeating the optimization process with different random initial points results in relatively low variations in the values of the optimal cost function (see Figure 5). The range of changes of the optimal cost function values in the level-ground condition is 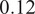 and in the downhill slope condition is 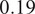 . These ranges are relatively low given that the improvements lower than 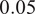 end the optimization process due to satisfying the convergence criteria. The stable cost function values span approximately 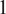 unit, ranging from 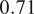 to 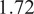 for level-ground gait, and around 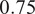 units, from 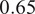 to 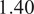 , for downhill slope gait. Therefore, the variation in optimal cost function values represents about 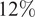 of the total range for level-ground condition values and 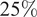 for downhill slope gait. Moreover, the median values of the optimal solutions in both conditions are close to the best solutions found, which means that it is very likely to reach a local minimum sufficiently close to the global optimum. Since the median of the 5 runs of the optimization used for the design of the exoskeleton is almost identical to the 20 runs used for the validation of the platform, it can be concluded that repeating the optimization for 5 times is sufficient to reach the optimal value. Additionally, the kernel density estimate of the 5 runs is close to the 20 runs, which means that the probability of achieving the same result is similar when the optimization is repeated 5 times compared to 20 times. Even though the optimization problem is non-convex and contains multiple local minima, the proposed optimization platform demonstrates the ability to achieve reasonable results with relatively few iterations. More specifically, the platform requires 160 iterations for level-ground and 83 iterations for downhill gait conditions across the 5 runs for each case. This is significantly fewer than the approximately 1300 combinations evaluated using the brute-force method in this study; a number that can increase dramatically with finer step sizes. Given that each predictive simulation in SCONE has a computational cost of approximately 5–10 min depending on the complexity of the movement (sloped gait usually takes longer than over-ground gait), the total time required for the brute-force evaluation of around 1300 parameter sets is in the order of 108–217 h. In contrast, our optimization platform achieves comparable results in approximately 9–17 h, representing a significant reduction in computational time. This advantage becomes even more critical when considering higher-dimensional design spaces, where exhaustive search methods would be computationally infeasible. Thus, the proposed method is computationally efficient and reliable in finding high-quality optima for both design problems, offering a significant advantage over brute-force approaches.

### 4.2. Optimization of the knee exoskeleton

The optimization results suggest that incorporating a damper into the knee joint during level-ground and downhill gait can effectively reduce the joint load at the knee without significantly increasing the load on the hip or ankle, the results of which are not presented in this article. This result agrees with our initial hypothesis that adding a damping element at the knee joint reduces the load on the biological joint. It is also observed that the amount of this reduction in load is higher for the downhill slope gait compared to the level-ground gait. Furthermore, the amount of the biological joint’s negative peak power is decreased in both gait conditions after the damper is added, which is in agreement with our initial hypothesis that the damper is dissipating energy instead of the biological joint in the Early Stance phase, in line with the findings of other studies (Xie et al., 2019). Moreover, the damper’s peak applied moment is less than half of the required biological knee moment in the baseline condition for both gait scenarios, which is a reasonable external torque to be applied to the human joint.

The passive exoskeleton beneficially reorganizes muscular forces and directly transfers load, resulting in reduced biological knee joint load and influencing metabolic cost. The knee joint load is a composite of muscle forces, body segment weights, GRFs, and critical interaction forces with the exoskeleton’s attachments on the femur and tibia. These direct interaction forces allow the device to share the biological joint’s burden, enabling the neuromuscular system to adopt new strategies. During level-ground walking, muscle force changes are not uniform; some muscles decrease while others increase. This reflects a coordinated adjustment where reductions in key knee muscles offload the joint, while increases may maintain dynamic stability or compensate for the device, ultimately achieving a 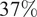 knee load reduction. The muscle forces during downhill walking show a more consistent trend towards force reduction for muscles crossing the knee. This broader reduction in muscular forces around the knee aligns with the more substantial decrease in biological knee joint load ( 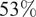 ) seen in this condition. Therefore, the observed shifts in muscle force patterns, even those involving increased activity, enable the exoskeleton to reduce net biological knee joint load. This interplay of load transfer and strategically altered muscle recruitment confirms the device’s potential to reduce forces on the knee joint while maintaining overall gait function, as reflected in changes to metabolic cost and our predictive simulation’s optimizations.

To provide additional support to our optimal design solution and the predicted biomechanical outcomes, we draw qualitative comparisons with experimental observations from other studies on knee exoskeletons. For instance, the observed reduction in biological knee torque and power in our simulations aligns well with the experimental results of Xie et al. (2019), which report similar beneficial effects with a knee-braced energy harvester. While our predicted knee load reductions are substantial ( 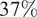 for level-ground and 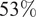 for downhill gait), potentially overestimating the magnitude compared to the 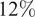 reduction of the impact forces on the user’s knee noted by Alvarado-Rivera et al. (2022) with their semi-active knee orthosis, the qualitative agreement in the direction of the desired effect remains consistent. Such over-estimations in magnitude are acknowledged in the predictive simulation literature (Franks et al., 2020; Jin et al., 2024), but the ability of our framework to identify beneficial trends is crucial for initial design guidance. This qualitative alignment with experimental findings from other exoskeleton designs provides further support for the plausibility of our simulation results.

The results of the optimization for the two movement scenarios show that the same clutch mechanism can be considered in the optimal design of the exoskeleton. The optimization process proposes a knee damper exoskeleton that is only active during the Stance phase of both gait scenarios. Therefore, a mechanical clutch system similar to the proposed design of Yandell et al. (2019) can be an option for the first prototype of the exoskeleton. On the other hand, the optimization process concludes that the damping coefficient of the device’s knee damper needs to be adjustable to have an optimal effect on level-ground and downhill gait. Although the damper is not required to change its damping characteristics during the movement, the optimal damping required for each movement scenario is different. Accordingly, semi-active systems such as adjustable hydraulic dampers (Naseri et al., 2020) and Magnetorheological (MR) dampers (Bhat et al., 2023), which are becoming more popular for wearable assistive devices, could be applied to implement our designed system. MR dampers with controllable smart materials through adjusting the magnetic field (Liu et al., 2022) have been used in the design of knee exoskeletons since they can function as brakes or clutches in different movement phases (Chen and Liao, 2006). The viscosity of MR dampers changes under a magnetic field, commonly adjusted in knee exoskeletons by altering the coil’s electrical current to modify the magnetic flux density and damping characteristics (Chen et al., 2017; Liu et al., 2022). Alternatively, a separate motor can displace a permanent magnet to achieve similar changes (Song et al., 2023).

### 4.3. Limitations and future directions

The main limitation of this study is that the predictive simulation results provide estimates of the expected outcomes, which require validation through experimental studies with the developed exoskeleton prototype. The potential for differences between simulated and real-world outcomes, as noted in the literature by Franks et al. (2020), necessitates this crucial next step. While the simplified biomechanical model used for forward simulation cannot fully replicate biological complexity, it still demonstrates sufficient accuracy in predicting movement dynamics. For example, minor discrepancies in baseline GRFs are primarily attributed to the simplified foot-ground contact model (De Groote and Falisse, 2021). Despite this, the framework reliably identifies optimal design parameters that align with expected biomechanical trends.

Another challenge is the sensitivity of the forward simulation method to small parameter changes, which can lead to variations in the optimal neuromuscular controller parameters (Firouzi et al., 2024). To mitigate this, we constrain the search space of the neuromuscular controller parameters, ensuring stability and consistency across similar conditions. While this approach reduces drastic parameter shifts, it also slightly restricts the flexibility of the optimization algorithm in finding a stable solution for a wide range of device parameters. However, this trade-off is acceptable, as it supports the assumption that biological feedback gains remain relatively stable across comparable scenarios. Additionally, while the non-convexity of the design space may increase optimization complexity, the framework remains effective in navigating these challenges to reach practical design solutions.

Future research can investigate the sensitivity of the optimal exoskeleton design to different weightings in the cost function or explore a multi-objective optimization approach to generate a Pareto front of solutions representing the trade-off between knee load reduction and cost of transport. In that case, the optimization platform can suggest a range and the optimized values can be selected by the human-in-the-loop optimization (Zhang et al., 2017), concerning other factors (e.g., user comfort). Furthermore, to enhance the reliability and applicability of the proposed framework, our next steps will focus on developing a prototype of the optimized exoskeleton design. This prototype will be tested in experimental studies across various movement scenarios, such as level-ground and sloped gait, to validate the simulation outcomes and assess the device’s performance in real-world conditions. These experiments will provide critical insights into the accuracy of predicted kinematics and kinetics behavior, and can be utilized in improving the models used for the next iterations of the design process via the optimization framework.

## 5. Conclusion

The proposed design framework for assistive wearable devices performs well in finding an optimal solution. It serves as the initial design for the first prototype of the device, even though it might not be the global optimum solution due to the limitations of the models used in the framework. The design of a knee exoskeleton, which is used in this study to illustrate the performance of the proposed framework, shows that the presented approach is quite robust in finding an optimal solution in different movement scenarios. The value of the proposed method becomes more evident when the number of design parameters increases, which makes the investigation of all candidate parameters computationally expensive. The optimization process is also able to give insight into the type of design parameters needed for an optimal device in more than one movement scenario. In our design question, for instance, it is concluded that the clutch mechanism does not need to be adaptable, whereas the damper needs to be adjustable. This information helps the designer make decisions based on the insights gained from biomechanical simulations in the initial phases of the design process.

## Data Availability

The data that support the findings of this study are available from the corresponding author, Asghar Mahmoudi, upon reasonable request.
